# Antibody Responses to Trivalent Inactivated Influenza Vaccine in Health Care Personnel Previously Vaccinated and Vaccinated for The First Time

**DOI:** 10.1038/srep40027

**Published:** 2017-01-18

**Authors:** Kuan-Ying A. Huang, Shih-Cheng Chang, Yhu-Chering Huang, Cheng-Hsun Chiu, Tzou-Yien Lin

**Affiliations:** 1 Division of Infectious Diseases, Department of Pediatrics, Chang Gung Children’s Hospital, Chang Gung Memorial Hospital, Taoyuan, Taiwan; 2 Molecular Infectious Disease Research Centre, Chang Gung Memorial Hospital, Taoyuan, Taiwan; 3 Department of Laboratory Medicine, Chang Gung Memorial Hospital, Taoyuan, Taiwan; 4 College of Medicine, Chang Gung University, Taoyuan, Taiwan; 5 Ministry of Health and Welfare, Taipei, Taiwan

## Abstract

Inactivated influenza vaccination induces a hemagglutinin-specific antibody response to the strain used for immunization. Annual vaccination is strongly recommended for health care personnel. However, it is debatable if repeated vaccination would affect the antibody response to inactivated influenza vaccine through the time. We enrolled health care personnel who had repeated and first trivalent inactivated influenza vaccination in 2005–2008. Serological antibody responses were measured by hemagglutination-inhibition (HI) test. Subjects with repeated vaccination had higher pre-vaccination and lower post-vaccination HI titer than those with first vaccination, although serological responses between groups might vary with different antigen types and while the drifted strain was introduced in the vaccine. Higher fold rise in the HI titer was observed in the group with first than repeated vaccination and the fold increase in the HI titer was inversely correlated with pre-vaccination titer in 2007 and 2008. Nevertheless, no significant difference in the day 28 seroprotection rate was observed between groups with repeated and first vaccination in most circumstances. Further studies are needed to understand the long-term effect of repeated vaccination on the antibody response both at the serological and repertoire levels among health care personnel.

Influenza A H1N1, A H3N2 and B viruses circulate in humans and cause annual epidemics around the world^1^. Each year, seasonal influenza infections lead to an estimate of 250,000–500,000 deaths^2^. Administration of influenza vaccine is one effective measure to prevent infections and severe illnesses^1^,^3^. Protection against influenza by inactivated vaccine is primarily mediated by virus-specific antibody response in humans^4^,^5^. Viral envelope hemagglutinin (HA), the primary target for vaccine-induced antibody response, is responsible for viral attachment to the host cell and subsequent fusion process^6^. Serological HA-specific antibody level is commonly measured by the hemagglutination inhibition (HI) test and the HI titer is generally used to validate the immunogenicity of inactivated influenza vaccine^7^,^8^.

HA-specific antibody response to inactivated influenza vaccination is mainly strain-specific^8^. Low fidelity of viral RNA-dependent RNA polymerase results in continuous accumulation of point mutations on the HA glycoprotein^9^. Mutations of viral HA antigen are associated with the emergence of drifted strains, to which previously vaccinated individuals might either lack or have insufficient antibody immunity^7^,^10^,^11^. A constant update of antigen components in the vaccine is therefore required to provide the prompt protection. Moreover, vaccine-induced serological titer might decay with the time and fail to achieve the protective level in the oncoming influenza season^7^,^8^,^12^. Thus, annual influenza vaccination remains the most important strategy for high-risk populations, such as pregnant women, young children, people with underlined diseases, and health care personnel, to maintain protective antibody immunity^13^.

Health care personnel (HCP) have a high risk of exposure to influenza viruses in their working environment and outbreaks of influenza in hospitals have been described^14^,^15^,^16^. Evidence shows that vaccination of HCP might reduce influenza transmission in health care settings, staff sickness and absence, and influenza-associated mortality and morbidity among individuals at increased risk for severe illnesses^17^. Several campaigns have been undertaken to improve the influenza vaccination coverage among HCP^16^,^17^,^18^. While the health care worker receives inactivated influenza vaccination, it is expected that an individual with prior vaccination would generate stronger strain-specific antibody response than those without prior one due to a recall of humoral immunological memory^19^,^20^. However, it has been reported that repeated influenza vaccinations might be associated with reduced serological antibody response and decreased vaccine effectiveness^12^,^21^,^22^,^23^,^24^,^25^.

To investigate the antibody response to trivalent inactivated influenza vaccine (TIV) and the effect of repeated vaccination on the antibody response among HCP, a convenience sample of consenting health care personnel at Chang Gung Memorial Hospital, Taiwan were enrolled in 2005–2008. Serum antibody titer to influenza vaccine antigens was measured before and 4 weeks after vaccination by hemagglutination-inhibition test.

## Results

A total of 113 HCP were enrolled and received annual TIV during the study period. Enrolled subjects could be classified into four groups (Table 1). Group 1 subjects were enrolled in 2005, had history of annual influenza vaccination prior to the study, and received annual TIV vaccinations from 2005 to 2008 (Table 2). Group 2 subjects were enrolled in 2006, received first TIV vaccination in October 2006, and further vaccinations of 2007/08 and 2008/09 TIVs. Group 3 subjects were enrolled in 2007, received first TIV vaccination in October 2007, and further vaccination of 2008/09 TIV. Group 4 subjects were enrolled in 2008 and received first TIV vaccination in October 2008. 11 of 18 (61%) subjects in the Group 1 were senior health care workers. In contrast, 23 of 25 (92%) subjects in the Group 2, 33 of 35 (94%) subjects in the Group 3, and 33 of 35 (94%) subjects in the Group 4 were clinical clerks. The mean age of Group 1 is significantly higher that that of other three groups. No significant difference in the mean age was observed among Groups 2, 3 and 4.

### Higher pre-vaccination and lower post-vaccination hemagglutination-inhibition titer in the repeated- than first-vaccination groups

Prior to vaccination, HI titer against the H1 antigen was detectable in all groups. Repeated-vaccination groups had higher pre-vaccination titer against the H1 antigen than first-vaccination group in 2007 and 2008. No significant difference in pre-vaccination titers against H1 was found among groups with repeated vaccination Table 3.

Pre-vaccination titers against H3 and type B antigens were significantly higher in repeated-vaccination groups compared with first-vaccination group in 2006 and 2007. In 2008, among repeated-vaccination groups, only Group 1 had significantly higher pre-vaccination titers against H3 Brisbane07 and type B Florida06 than first-vaccination Group 4.

At day 28 after vaccination, elevated HI titer against the H1 antigen was detected in all groups, although Group 1 produced relatively low titers of HI antibodies (less than 40) against the H1 antigen in 2005, 2006 and 2008. The first-vaccination group generated a higher titer against the H1 antigen than repeated-vaccination groups through the study. Among repeated-vaccination groups, in 2007, Group 2 (2^nd^-time TIV) had a significantly higher titer than Group 1 (multiple TIV), but in 2008, no significant difference in the day 28 HI titers against H1 was noted.

For H3 antigens, no significant difference in day 28 HI titers was found among all groups through the study. No significant difference in day 28 HI titers against type B antigens was noted either among all groups in 2006. In 2007, no significant difference in day 28 HI titers against type B antigens was noted between Group 2 (2^nd^-time TIV) and Group 3 (1^st^-time TIV). In 2008, the first-vaccination group had a higher titer against type B Florida06 than repeated-vaccination Groups 2 and 3, but lower than Group 1 (Table 3).

### Higher fold rise in the hemagglutination-inhibition titer in the group with first than repeated vaccination

**Figure 1:**
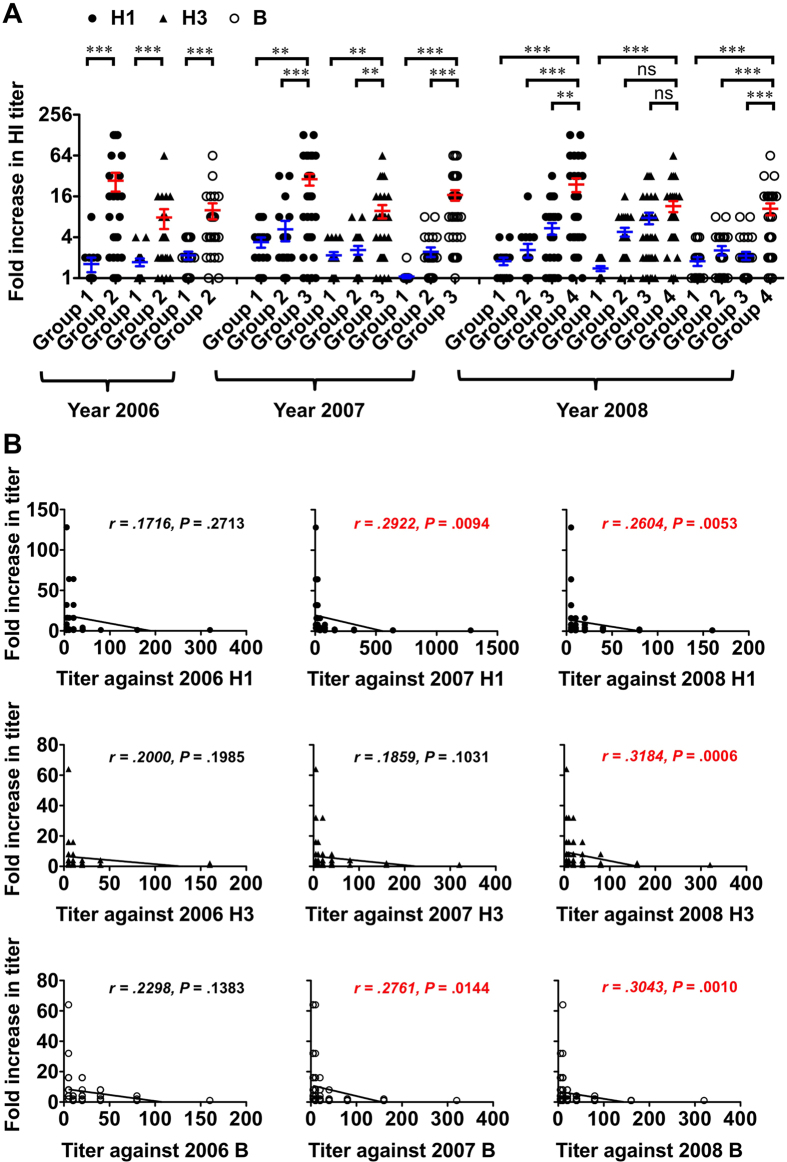
Analysis of the fold increase in HI titer upon TIV vaccination between groups. (**A**) Comparison of the fold increase in HI titer at 28 days after TIV vaccination to baseline between groups with first and repeated vaccination. The bar indicates the mean value ± standard error of the mean. The data for group with first vaccination were shown in red and those for groups with repeated vaccination shown in blue. Difference in the fold increase in HI titers between two groups was analyzed by Mann-Whitney test. *P < 0.01, **P < 0.001, ***P < 0.0001. (**B**) Analysis of the fold increase in HI titer at 28 days after TIV vaccination to baseline and the baseline HI titer against the vaccine antigen. Correlation was run between the fold increase in HI titer and pre-vaccination HI titer by Spearman rank correlation analysis. Abbreviations: ns, non-significant.

For H1 antigens, the fold increase in the HI titer was significantly higher in the first- than repeated-vaccination groups through the study (Fig. 1A). The average fold increases were 16 times higher in 2006, 5 to 8 times higher in 2007, and 4 to 13 times higher in 2008, for the first- than repeated-vaccination groups.

Higher fold increases in the HI titer against H3 and type B antigens were also noted in the first- than repeated-vaccination groups and the difference was statistically significant. For H3 antigens, the average fold increases were 5 times higher in 2006, 4 to 5 times higher in 2007, and 2 to 8 times higher in 2008; for type B antigens, the average fold increases were 5 times higher in 2006, 7 to 15 times higher in 2007, and 4 to 6 times higher in 2008, for the first- than repeated-vaccination groups.

The fold increase in the HI titer upon vaccination was inversely correlated with pre-vaccination titer in 2007 and 2008 (Fig. 1B). However, in 2006, the association between lower pre-vaccination titer and higher fold increase in the HI titer upon vaccination did not reach statistical significance.

### Similar post-vaccination seroprotection rate between repeated- and first- vaccination groups

No significant difference in the seroprotection rates against the H1 antigen was noted among groups prior to the vaccination in 2006. In 2007, among repeated-vaccination groups, Group 2 but not Group 1 had a higher pre-vaccination seroprotection rate than Group 3 who would have first TIV vaccination. In 2008, pre-vaccination seroprotection rate (11%) in the group with first vaccination was lower but the difference among groups was marginal Table 4.

At day 28 after vaccination, the seroprotection rate against the H1 antigen reached over 70% in repeated- and first-vaccination groups except Group 1. Group 1 (multiple TIV) had substantially lower day 28 seroprotection rate in 2006 (39%) and 2008 (56%) than other groups. In 2007, no significant difference in day 28 seroprotection rate against H1 was found among groups. In 2008, no significant difference in day 28 seroprotection rate was noted among repeated-vaccination groups (Group 2 and 3) and first-vaccination group (Group 4).

For H3 and B antigens, lower pre-vaccination seroprotection rates were noted in the group with first vaccination. After vaccination, the seroprotection rate against the H3 antigen was either nearly or over 70% both in repeated- and first-vaccination groups and no significant difference was found among groups through the study. The day 28 seroprotection rate against type B antigen was nearly or over 70% and was not significantly different among all groups in 2006 and 2007. In 2008, a significant difference in the seroprotection rate against type B antigen was noted among groups but both repeated- and first-vaccination groups had rates of nearly or over 70% (Table 4).

## Discussion

This study demonstrated that administration of inactivated influenza vaccines induced strain-specific hemagglutination-inhibition antibody titers and day 28 seroprotection rates against the vaccine antigen both in the health care personnel with first and repeated vaccination.

In the study, hemagglutination-inhibition assay was used to measure HA head-specific antibody responses to trivalent inactivated influenza vaccinations. Repeated-vaccination subjects possessed a substantial baseline HI antibody level prior to vaccination even though vaccine components were replaced by new variants (i.e. H1 antigens in 2007/08 and 2008/09, H3 antigen in 2006/07, or B antigens in 2006/07 and 2008/09 TIVs), suggesting the presence of cross-reactive HA head-specific antibody response in the humoral memory. Previously, we have demonstrated a subset of H1 head-specific antibodies induced by inactivated influenza vaccination could neutralize and cross-react to past seasonal H1 strains^11^. Cross-reactive HA-specific antibodies might target the conserved region near the receptor-binding site of hemagglutinin head, although the majority of HA cross-reactive antibodies would recognize the stem region^26^,^27^,^28^,^29^. Other conserved neutralizing epitopes on the H3 HA head have also been described^30^,^31^. Nevertheless, it is unclear about the protective level of these cross-reactive HA-specific antibodies and the declining trend of the HI titer with the time within previously vaccinated individuals. The result showed that the antibody titer did not achieve a protective level (HI titer ≥40) against drifted strains in most subjects with previous TIV vaccination until they were boosted with annual vaccination in the study. It seems that annual influenza vaccination is still necessary for the establishment of antibody immunity and prevention from influenza infections, especially while the drifted strain emerges.

Antigen components in the TIVs have been updated during the study and we note that while the new antigen was introduced, individuals with repeated vaccination could still generate a high HI titer against the updated antigen. The H1 vaccine antigen remained unchanged in 2002–2006, but in 2007, New Caledonia99 strain was replaced with Solomon Islands06 strain in the 2007/08 TIV. Upon 2007/08 TIV vaccination, all three groups in the study, in which Group 1 and Group 2 were previously vaccinated and Group 3 received their first TIV, had strong HI titers against Solomon Islands06, although Group 3 had the highest antibody titer and higher fold increase in HI titer than other groups. In 2008, the H3 antigen was replaced with the drift variant Brisbane07 strain in the 2008/09 TIV and groups with repeated vaccination produced a similar HI titer against Brisbane07 antigen as the group with first vaccination. In a previous study, Sasaki *et al*. showed that young adults with prior TIV vaccination in 2004 had non-inferior fold increase of HI antibody response to updated H3 California04 antigen in 2005 as those without prior year vaccination^32^. A strong antibody response to antigenically novel antigens in the TIV might result from a robust antigen-specific B cell response among those individuals with repeated vaccination^33^. In addition, while adults who have the first TIV produce a higher HI titer to the updated vaccine antigen than those with repeated TIV, the HI antibody persistence rate was found to be similar between both groups in a two-year follow-up study^12^.

The antigen component might remain the same as those included in previous year TIV while there were no significant drifts in the hemagglutinin and neuraminidase antigens of circulating strains and no significant HI fold decrease in the post-infection ferret antisera. For instance, in the study, H3 and type B antigens were Wisconsin05 and Malaysia04 in both 2006/07 and 2007/08 TIVs. It was expected that, with the presence of immunological memory, subjects who had previous 2006/07 TIV vaccination would produce stronger antibody responses to the same H3 and type B antigens than those with first TIV vaccination in 2007. However, no significant difference in the HI titers against 2007/08 TIV H3 and type B antigens was found between first- and repeated- vaccination groups. It has been unclear about how the context of influenza virus-specific immunological memory affects vaccine-induced antibody repertoire of an individual in a long period of time. A follow-up study found that while experiencing repeated exposure to the same antigen in the TIVs, lower baseline HI titer is associated with a stronger antibody-secreting and memory B cell response and serological antibody titers to subsequent vaccination^33^. Beyer *et al*. also reported that previous vaccination history might have a significantly negative association with post-vaccination HI titer to TIV in young adults^34^. Nevertheless, controversial results showed similar post-vaccination HI and IgG antibody response to inactivated vaccines regardless of the vaccination history^35^. It is proposed that pre-existing antigen-specific antibodies might mask the presentation of viral epitopes and subsequently reduce the magnitude and breadth of secondary antibody response to repeated influenza exposure. Moreover, a phenomenon, original antigenic sin (OAS), is described in relation to influenza virus-specific immune response, although the underlining mechanism is undetermined. Generally, this phenomenon states that prior (original) exposure to an influenza antigen would lead to a subsequent suboptimal immune response to a related antigen and is demonstrated in ferrets with sequential influenza infections^36^,^37^. When OAS occurs in the host with recent infection, due to absent or diminished antibody response against the latest strain, the infected host may therefore develop severe illness. Kim *et al*. showed that mice previously infected with H1N1 A/Puerto Rico/8/1934 virus produced significantly impaired antibody response to recent infection of H1N1 A/Fort Monmouth/1/1947 virus and developed high viral load in lungs after challenge^38^. Original antigenic sin responses to influenza virus seem to be observed mainly in the circumstance of live infections. Whether or not this phenomenon exists in human with sequential inactivated influenza vaccination is still debatable^39^,^40^.

In the study, Group 1 subjects produced relatively poor HI antibody responses against H1 antigens and their day 28 seroprotection rates against H1 were below 60% in 2005, 2006, and 2008. In 2006, Group 1 (multiple TIV) had significantly lower HI titer and day 28 seroprotection rate than Group 2 (first TIV). It has to be mentioned that Group 1 has a higher average age than any of other groups. Reduced HI antibody response, lower frequency of vaccine-specific antibody-secreting cells, and decreased memory B cell to plasma cell differentiation have been described in elderly people aged 60 years or over when compared to young and middle-aged adults after receiving seasonal influenza vaccine^35^,^41^,^42^. Nevertheless, the average age of Group 1 was 34.2 ± 9.2 years, seven subjects of Group 1 aged between 21 to 30 years, six aged between 31 to 40 years, four aged between 41 to 50 years and only one aged 57 years. Aging might just partially explain unsatisfactory outcome of repeated TIV vaccination in the Group 1 in specific years. Confounding factors such as past exposure history to live influenza virus and vaccine antigens might affect the immune response to recent TIV vaccination in senior health care workers although the precise mechanism has not been established yet.

Previous immune memory to HA antigens might have profound influence to the specificity and the breadth of HA-specific antibody response in humans. Andrew *et al*. have shown that upon vaccination with H1N1 A/California/07/2009 antigen, those with high pre-existing California09 HA-specific antibody titer generated HA head-biased antibody-secreting B cell response; in contrast, those producing broadly reactive HA stem-biased B cell response had a significantly lower pre-existing antibody titer^43^. This indicates that HA head immunodominant epitopes recognized by pre-existing memory B cells might preferentially be activated while encountering particular antigen. In the study, a significant rise of strain-specific HI titer was detected in both repeated- and first-vaccinated groups, indicating the induction of HA head-specific antibody response upon inactivated influenza vaccination. At the serological level, we also noted that higher day 28 HI titers against the H1 antigen and fold increase in the HI titer within first- than repeated-vaccination groups. However the information about the breadth and specificity of HA-specific antibody repertoire was lacking and the magnitude of hemagglutinin stem-specific antibody response to TIV vaccinations was unclear in the study.

Since hemagglutination inhibition test is the only method we applied to evaluate the antibody response to inactivated trivalent influenza vaccination in the study, neuraminidase-specific serological response is not assessed here. Neuraminidase (NA) is another major surface antigen in the inactivated influenza vaccine in addition to hemagglutinin. An obvious increase of neuraminidase-inhibition (NI) antibody titer could be detected 28 days post-vaccination using five commercially available TIVs and the NI titer significantly correlated with the HI titer both in the pre- and post-vaccination time points^44^. Although dominant antibody response to HA over NA antigens has been observed upon inactivated influenza vaccination, undetermined NA amounts in the vaccines and different assays used to measure functional NA-specific antibodies make it difficult to compare anti-HA with anti-NA responses among studies^44^,^45^,^46^,^47^,^48^. While HI antibody response is generally accepted as an immune marker of protection against influenza and used as the immunogenicity standard for licensure of inactivated vaccines, several assays to evaluate NA-specific antibodies are also developed. Plaque size-reduction assay can be used to assess the level of inhibition of viral replication by NA-specific antibodies but a standardized method is unavailable and the assay reproducibility between laboratories is questionable^49^,^50^. Enzyme-linked lectin-based assay is mostly used to examine functional NA-specific serological response; however, antigens such as purified protein, pseudotyped virus, or reassortant virus carrying novel HA to whom humans were naïve have to be prepared and this makes the assay difficult to be adapted for routinely screening large numbers of samples^44^,^51^,^52^. To establish a standardized, simple, and highly reproducible method to examine functional NA-specific serological response is desirable.

It has been shown that NA-specific antibodies contribute to reducing the illness severity and viral shedding^53^,^54^,^55^,^56^. NA-specific antibodies could play a significant role in the host immunity against influenza, while the drifted strain containing antigenic changes in the HA circulates or the host has insufficient serological HI titer against viruses. In the present study, we noted that repeated-vaccination groups generated a relatively lower HI titer against the H1 antigen than the first-vaccination group after TIV vaccination. The difference in NA-specific antibody response among groups is unclear. Previous studies showed that the antigenic competition between HA and NA proteins might be associated with a suppressed NA-specific immune response of primed hosts to whole virion antigens^46^,^57^. Kilbourne showed that a strong NI antibody response could be achieved when humans with preexisting memory were boosted with viral antigens containing the same neuraminidase but novel hemagglutinin^58^. Further study to measure functional NA-specific antibody responses to repeated TIV antigens and delineate the effect of immune memory to prior TIV antigens on the context of HA and NA-specific antibody responses to subsequent vaccination should be conducted.

Taken together, HA-specific serological response between groups with repeated and first TIV vaccination might vary with different antigen types and while the drifted strain is introduced in the vaccine and lower baseline HI titer was found to be correlated with higher fold increase in HI titer against the vaccine antigen. However, there was no significant difference in the day 28 seroprotection rates between groups with repeated and first vaccination in most circumstances. The influence of repeated vaccination on the immunogenicity and effectiveness of TIV in HCP should be further examined in the longitudinal and large-scale study in the near future.

## Methods

### Ethic statement

The study protocol and informed consent were approved by the ethics committee at the Chang Gung Medical Foundation Institutional Review Board. Each subject provided signed informed consent. The study and all associated methods were carried out in accordance with the approved protocol, the Declaration of Helsinki and Good Clinical Practice guidelines.

### Study design

Between October 2005 and December 2008, we conducted a prospective study to examine the antibody response to trivalent inactivated influenza vaccine among health care personnel previously vaccinated and vaccinated for the first time in Chang Gung Memorial Hospital, Taiwan. Health care personnel aged 18 to 64 years, were clinically healthy, and eligible to receive annual TIV vaccination were recruited. Those who had a history of laboratory-confirmed influenza infection and influenza-like illnesses within 3 months prior to enrollment or vaccination, had chronic cardiovascular or pulmonary illness, had autoimmune disorder, were immunosuppressive or immunocompromised, and had a history of allergy to the component of TIV were excluded. Influenza-like illness was defined as having been sick with fever of ≥38 °C and cough and/or sore throat.

A convenience sample of consenting health care personnel in Chang Gung Memorial Hospital were enrolled in 2005–2008. Age, gender, and history of TIV vaccination were recorded. Peripheral blood was collected prior to and 28 days after TIV vaccination.

### Vaccine

Enrolled subjects were vaccinated with TIV Fluarix (GlaxoSmithKline Biologicals, Dresden, Germany) in 2005, 2006 and 2007. In 2008, enrolled subjects were vaccinated with TIV KKB/KI-Flu (Adimmune Corporation, Taichung, Taiwan). The 0.5 mL inactivated split-virion vaccine contains 15 μg hemagglutinin antigens of each strain. Both vaccines in the study did not contain the adjuvant. Influenza viral antigens for 2005/06, 2006/07, 2007/08, and 2008/09 TIVs were listed in the Table 2.

### Hemagglutination inhibition test

Serum antibody titers were determined by hemagglutination-inhibition assay. Vaccine strains kindly provided by GSK Taiwan and human (O blood type) red blood cells were used in the HI assay. Pre- and post-vaccination sera from each subject were stored at −80 °C until tested. All sera were treated with receptor-destroying enzyme and then heat-inactivated at 56 °C for 30 min before use. A serial 2-fold dilution of sera was made from 1:10 to 1:1280. All sera were tested in duplicate.

### Definitions

The HI titer was defined as the reciprocal of the highest dilution of serum which completely inhibited hemagglutination. Serum HI titer of ≥40 was considered as seroprotective. Seroprotection rate was defined as the percentage of subjects with HI titer ≥40. Serum samples with HI titer <10 were assigned a titer of 5. The ratio of geometric mean titers from 28 days post-vaccination to pre-vaccination was defined as the fold increase of antibody titer.

### Statistical analysis

Graphs were presented by GraphPad Prism software and statistical analyses were performed by GraphPad Prism and SPSS. The antibody titer difference and the fold increase in antibody titers between two groups were analyzed by Mann-Whitney test. Kruskal-Wallis test with post-hoc Dunn’s multiple comparison was applied to analyze the difference in ages and antibody titers among three or more groups. The correlation between pre-vaccination antibody titer and the fold increase in the antibody titer was analyzed using Spearman rank correlation analysis. The P value of less than 0.05 was considered significant.

## Additional Information

**How to cite this article**: Huang, K.-Y. A. *et al*. Antibody Responses to Trivalent Inactivated Influenza Vaccine in Health Care Personnel Previously Vaccinated and Vaccinated for The First Time. *Sci. Rep.* **7**, 40027; doi: 10.1038/srep40027 (2017).

**Publisher's note:** Springer Nature remains neutral with regard to jurisdictional claims in published maps and institutional affiliations.

## Figures and Tables

**Table 1:** 113 health care workers enrolled in the study.

	Group 1 (n = 18)	Group 2 (n = 25)	Group 3 (n = 35)	Group 4 (n = 35)
Male:Female	8:10	17:8	22:13	21:14
Age (yrs)*	34.2 ± 9.2	24.3 ± 4.1	23.4 ± 1.5	23.6 ± 1.8
Previous influenza vaccination prior to enrollment	yes	none	none	none
Enrollment year	2005	2006	2007	2008
Northern Hemisphere’s TIV received during the study	2005/06, 2006/07, 2007/08, 2008/09	2006/07, 2007/08, 2008/09	2007/08, 2008/09	2008/09

*Age was presented as mean ± standard deviation. Post-hoc Dunn’s test following the Kruskal-Wallis test showed that Group 1’s mean age was significantly higher than that of other three groups (*P* < 0.0001).

Abbreviations: TIV, trivalent inactivated influenza vaccine.

**Table 2:** Viral antigens in the Northern Hemisphere’s trivalent inactivated influenza vaccine (TIV) in the study.

**2005/06 TIV**
A/New Caledonia/20/99(H1N1)-like virus (since 2000–01)
A/California/7/2004(H3N2)-like virus
B/Shanghai/361/2002-like virus (Yamagata lineage)
**2006/07 TIV**
A/New Caledonia/20/99(H1N1)-like virus
A/Wisconsin/67/2005(H3N2)-like virus
B/Malaysia/2506/2004-like virus (Victoria lineage)
**2007/08 TIV**
A/Solomon Islands/3/2006(H1N1)-like virus
A/Wisconsin/67/2005(H3N2)-like virus
B/Malaysia/2506/2004-like virus (Victoria lineage)
**2008/09 TIV**
A/Brisbane/59/2007(H1N1)-like virus
A/Brisbane/10/2007(H3N2)-like virus
B/Florida/4/2006-like virus (Yamagata lineage)

**Table 3:** Serological antibody titer at baseline and 28 days after vaccination.

GMT (95% CI)	Group 1	Group 2	Group 3	Group 4	P value*
**Years and H1N1 antigens**					
2005 (A/New Caledonia/20/1999-like virus)					
Pre	17 (10–26)	—	—	—	—
Post	25 (16–40)	—	—	—	—
2006 (A/New Caledonia/20/1999-like virus)					
Pre	15 (10–22)	13 (8–22)	—	—	0.2698
Post	19 (12–30)	103 (52–203)	—	—	0.0010
2007 (A/Solomon Islands/3/2006-like virus)					
Pre	38 (23–66)	100 (48–207)	25 (17–36)	—	0.0070 (0.0031)
Post	101 (71–144)	243 (159–370)	320 (238–430)	—	0.0001 (0.3087)
2008 (A/Brisbane/59/2007-like virus)					
Pre	22 (16–30)	22 (15–33)	19 (13–26)	8 (6–10)	<0.0001^#^ (<0.0001^#^)
Post	34 (23–52)	45 (30–66)	63 (49–82)	87 (60–126)	0.006 (0.0449)
**Years and H3N2 antigens**					
2005 (A/California/7/2004-like virus)					
Pre	17 (14–21)	—	—	—	—
Post	50 (30–86)	—	—	—	—
2006 (A/Wisconsin/67/2005-like virus)					
Pre	27 (18–41)	10 (7–15)	—	—	0.0004
Post	42 (26–66)	41 (27–62)	—	—	0.8589
2007 (A/Wisconsin/67/2005-like virus)					
Pre	31 (19–48)	26 (16–41)	9 (7–12)	—	<0.0001^#^ (0.0003)
Post	59 (35–99)	54 (34–86)	50 (33–75)	—	0.9471 (0.9575)
2008 (A/Brisbane/10/2007-like virus)					
Pre	101 (77–131)	19 (12–30)	19 (14–26)	10 (8–13)	<0.0001 (0.0116)
Post	132 (99–176)	70 (47–104)	83 (58–120)	74 (47–116)	0.1392 (0.6195)
**Years and B antigens**					
2005 (B/Shanghai/361/2002-like virus) (Yam)					
Pre	25 (17–37)	—	—	—	—
Post	38 (29–51)	—	—	—	—
2006 (B/Malaysia/2506/2004-like virus) (Vict)					
Pre	29 (19–45)	9 (7–12)	—	—	<0.0001
Post	54 (33–90)	53 (33–85)	—	—	0.7163
2007 (B/Malaysia/2506/2004-like virus) (Vict)					
Pre	42 (26–66)	25 (16–40)	8 (6–10)	—	<0.0001^#^ (<0.0001)
Post	43 (27–68)	49 (31–76)	82 (57–117)	—	0.0484 (0.0594)
2008 (B/Florida/4/2006-like virus) (Yam)					
Pre	90 (68–118)	21 (13–32)	30 (22–43)	15 (11–20)	<0.0001 (0.0164)
Post	137 (110–171)	41 (28–61)	53 (39–71)	94 (73–120)	<0.0001 (0.0016)

*Difference in antibody titers between two groups was analyzed by Mann-Whitney test. Kruskal-Wallis test was used to analyze the difference in antibody titers among three or more groups. The result of statistical analysis excluding the data of Group 1 was also provided in parentheses in view of the average age of Group 1 being higher than that of any other group.

^#^In post-hoc analysis of Kruskal-Wallis multiple comparison, serological titer in the group with first-time TIV vaccination is significantly different from that of other groups with repeated TIV vaccination.

Abbreviations: GMT, geometric mean titer; Yam, yamagata lineage; Vict, victoria lineage.

**Table 4:** Seroprotection rate at baseline and 28 days after vaccination.

Seroprotection rate (% of HI titer ≥40)	Group 1	Group 2	Group 3	Group 4	P value*
**Years and H1N1 antigens**					
2005 (A/New Caledonia/20/1999-like virus)					
Pre	22	—	—	—	—
Post	50	—	—	—	—
2006 (A/New Caledonia/20/1999-like virus)					
Pre	22	20	—	—	1.0000
Post	39	76	—	—	0.0259
2007 (A/Solomon Islands/3/2006-like virus)					
Pre	56	68	40	—	0.0968 (0.0396)
Post	94	96	100	—	0.4123 (0.4167)
2008 (A/Brisbane/59/2007-like virus)					
Pre	39	44	34	11	0.0286 (0.0140)
Post	56	72	89	86	0.0233 (0.2103)
**Years and H3N2 antigens**					
2005 (A/California/7/2004-like virus)					
Pre	11	—	—	—	—
Post	61	—	—	—	—
2006 (A/Wisconsin/67/2005-like virus)					
Pre	44	12	—	—	0.0312
Post	61	64	—	—	1.0000
2007 (A/Wisconsin/67/2005-like virus)					
Pre	44	40	9	—	0.0041 (0.0090)
Post	72	68	71	—	0.9440 (0.7832)
2008 (A/Brisbane/10/2007-like virus)					
Pre	100	36	34	14	<0.0001 (0.0910)
Post	100	80	89	77	0.1273 (0.4365)
**Years and B antigens**					
2005 (B/Shanghai/361/2002-like virus) (Yam)					
Pre	50	—	—	—	—
Post	78	—	—	—	—
2006 (B/Malaysia/2506/2004-like virus) (Vict)					
Pre	44	12	—	—	0.0312
Post	78	64	—	—	0.5027
2007 (B/Malaysia/2506/2004-like virus) (Vict)					
Pre	66	36	6	—	<0.0001 (0.0051)
Post	72	60	83	—	0.1436 (0.0751)
2008 (B/Florida/4/2006-like virus) (Yam)					
Pre	100	40	54	29	<0.0001 (0.0908)
Post	100	64	80	94	0.0028 (0.0126)

*Difference in seroprotection rates between two groups was analyzed by Mann-Whitney test. Kruskal-Wallis test was used to analyze the difference in seroprotection rates among three or more groups. The result of statistical analysis excluding the data of Group 1 was also provided in parentheses in view of the average age of Group 1 being higher than that of any other group.

Abbreviations: HI, hemagglutination-inhibition; Yam, yamagata lineage; Vict, victoria lineage.
